# Effect of metabolic health and obesity on all-cause death and CVD incidence in Korean adults: a retrospective cohort study

**DOI:** 10.1038/s41598-022-27097-8

**Published:** 2023-01-12

**Authors:** Ye-Seul Kim, Sang-Jun Shin, Yonghwan Kim, Joungyoun Kim, Hee-Taik Kang

**Affiliations:** 1 Department of Family Medicine, Chungbuk National University Hospital, 776 1-Soonwhan-ro, Seowon-gu, Cheongju, 28644 Republic of Korea; 2 Biostatistics Collaboration Unit, Department of Biomedical Systems Informatics, Yonsei University College of Medicine, 50-1 Yonsei-ro, Seodaemun-gu, Seoul, 03722 Republic of Korea; 3 College of Nursing, Mo-Im Kim Nursing Research Institute, Yonsei University, 50-1, Yonsei-ro, Seodaemun-gu, Seoul, 03722 Republic of Korea; 4 Department of Artificial Intelligence, University of Seoul, 163 Seoulsiripdae-ro, Dongdaemun-gu, Seoul, 02504 Republic of Korea; 5 Department of Family Medicine, Chungbuk National University College of Medicine, 1 Chungdae-ro, Seowon-gu, Cheongju, 28644 Chungbuk Republic of Korea

**Keywords:** Cardiology, Endocrinology, Risk factors

## Abstract

This study aimed to investigate the risk of all-cause mortality and incidence of CVD according to metabolic health and body mass index (BMI) in Korean adults. This study was retrospectively designed using the National Health Insurance Service-National Health Screening Cohort data. Participants were divided into six groups according to two category of metabolic syndrome and three categories of BMI. Hazard ratios (HRs) and 95% confidence intervals (CIs) for the composite outcome (all-cause mortality and incidence of CVDs) were estimated using multivariable Cox proportional hazards regression models. 151,706 participants aged ≥ 40 years were enrolled; median follow-up period was 9.7 years in the study. Compared to metabolically healthy normal weight, the fully adjusted HRs (95% CIs) of metabolically healthy overweight, metabolically healthy obese, metabolically unhealthy normal weight, metabolically unhealthy overweight, and metabolically unhealthy obese for composite outcome were 1.07 (1.03–1.12), 1.12 (1.07–1.17), 1.33 (1.25–1.41), 1.28 (1.22–1.34), and 1.31 (1.26–1.37), respectively, in men, and 1.10 (1.05–1.16), 1.22 (1.16–1.29), 1.34 (1.26–1.43), 1.27 (1.19–1.34), and, 1.40 (1.34–1.47), respectively, in women. High BMI and metabolic unhealthiness were associated with an increased risk on the composite of all-cause mortality and incidence of CVD in both sexes.

## Introduction

Cardiovascular diseases (CVDs), primarily ischemic heart disease (IHD) and stroke, are the leading cause of mortality and a significant contributor to disability. The global prevalence of CVD nearly doubled from 271 to 523 million from 1990 to 2019, and global trends for disability-adjusted life years and years of life lost also increased^1^.

There are already well-known causes of CVDs, including tobacco smoking, obesity, and several chronic diseases, such as hypertension and dyslipidemia^2^. Central obesity and metabolic syndrome (MetS) share a common metabolic abnormality: insulin resistance^3^. Insulin resistance, characterized by diminished tissue sensitivity to insulin, contributes to CVD development as a vascular risk factor; resulting from abnormalities of glucose metabolism, causing atherogenic dyslipidemia, blood pressure elevation, abnormal vascular reactivity, endothelial dysfunction, and chronic subclinical inflammation^4^. To prevent CVDs, reducing body weight in individuals with overweight or obesity, changing to healthier lifestyles in individuals with unhealthy behaviors, and controlling chronic diseases are necessary^5^. The results from several studies to investigate the effect on CVD or mortality of obesity or metabolic healthiness were inconsistent^6–8^; the “obesity paradox,” which is mortality decreases in obese populations rather than in normal-weight or risk of CVD incidence in metabolically unhealthy normal-weight increased as high as in the metabolically healthy obese population.

Obesity and MetS in Korean adults have steadily increased since 2005‒2006^9^. Peculiarly, these increasing trends in obesity and MetS are prominent in men^10^. Also, with the rising prevalence and consequence of mortality and disability with a heavy economic burden from CVDs, the importance of managing these modifiable risk factors to prevent CVDs and reduce deaths is increasing^11^. Still, no large-scale studies have been conducted to determine which of these two contributes more to all-cause mortality and incidence of CVD. It is necessary to re-evaluate the association between obesity, metabolic healthiness, and CVD outcome, but Korean studies are lacking.

This study aimed to investigate the association between risk factors such as obesity and MetS risk of all-cause mortality and CVD incidence according to metabolic health and BMI in Korean adults using data from the National Health Insurance Service (NHIS)-Health Screening (HEALS) Cohort.

## Material and methods

### Study participants

This study was analyzed based on the Korean NHIS-HEALS cohort database, which consisted of individuals randomly sampled 10% of the total population who had undergone the national health screening programs (NHSPs) between 2002 and 2003. All individuals included in the database were aged between 40 and 79 years in 2002, followed up through 2019. The cohort data contain death, healthcare usage, and health screening information. The variables from the NHIS were income-based insurance premium (a proxy for house income), demographic variables, date of death, cause of death, prescription records, and disease diagnosis codes. Blood pressure, anthropometric measurements, lifestyles (cigarette smoking, alcohol drinking, and physical activity), personal/family medical histories, and laboratory results were from the NHSPs. Korean NHIS provides NHSPs biennially. This cohort followed from 2002 to 2019; however, NHSPs measured lipid profile parameters such as serum triglyceride (TG), HDL cholesterol (HDL-C), and low-density lipoprotein cholesterol (LDL-C) levels since 2009; therefore, we set 2009 and 2010 as the baseline, and followed up to 2019.

**Figure 1:**
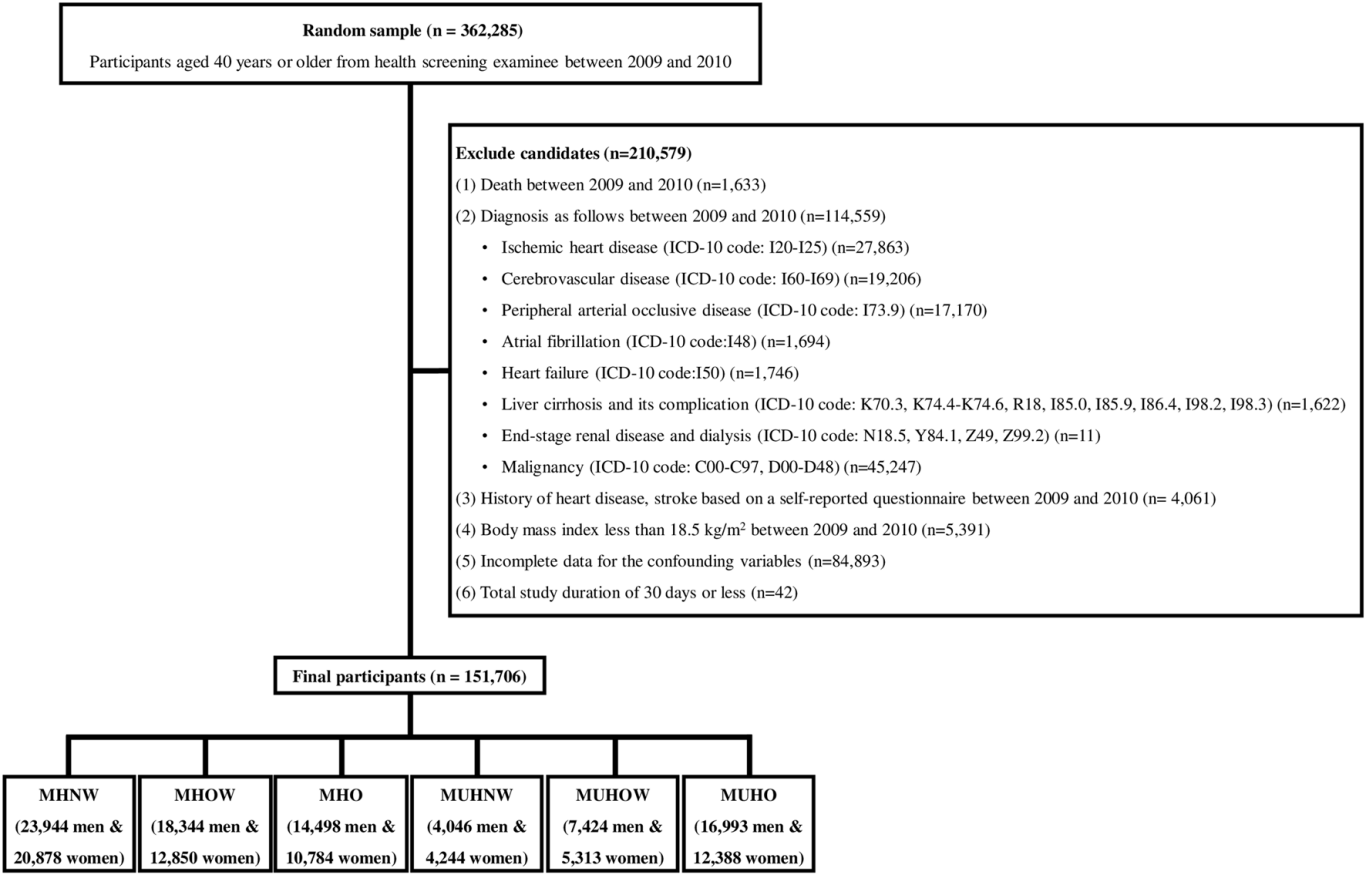
Flow chart of inclusion and exclusion criteria of participants.

Figure 1 presents this study's inclusion and exclusion criteria. Of the initial 362,285 participants at baseline (2009 and 2010), 210,579 individuals were excluded based on the following exclusion criteria: (1) participants who died between 2009 and 2010 (n = 1633); (2) those who were diagnosed with ischemic heart diseases (IHD) (I20–I25), cerebrovascular diseases (CbVDs) (I60–I69), peripheral arterial occlusive disease (I73.9), or atrial fibrillation (I48), heart failure (I50), liver cirrhosis and its complications (K70.3, K74.4-K74.6, R18, I85.0, I85.9, I86.4, I98.2, I98.3), end-stage renal disease and dialysis (N18.5, Y84.1, Z49, Z99.2), or malignancy (C00–C97, D00–D48) based on the 10th edition of the International Classification of Diseases (ICD-10) between 2009 and 2010 (n = 114,559); (3) those who reported heart disease or stroke in a self-reported questionnaire between 2009 and 2010 (n = 4061); (4) those who had a BMI < 18.5 kg/m^2^ between 2009 and 2010 (n = 5391); (5) those who had incomplete data for the confounding variables (n = 84,893); (6) those who had a total study duration ≤ 30 days (n = 42). Participants were sequentially excluded based on the criteria mentioned above, which were not mutually exclusive. After full exclusion, 151,706 participants (85,249 men and 66,457 women) were included in the final analysis.

The ethics committee of NHIS waived the need for informed consent because the data from the NHIS-HEALS were anonymized at all stages, including during data cleaning and statistical analysis. The Institutional Review Board of the Chungbuk National University Hospital approved the present study (CBNUH-2022-01-039), which adhered to the principles of the Declaration of Helsinki (1975).

### Definitions of study groups and covariates

Participants were divided according to the presence of MetS and BMI categories for each sex. Based on the modified Asian criteria from the American Heart Association/National Heart, Lung, and Blood Institute (AHA/NHLBI)^12^, MetS was defined as three or more of the following criteria: (1) waist circumference ≥ 90 cm (men) or 85 cm (women), (2) systolic blood pressure (SBP) ≥ 130 mmHg and/or diastolic blood pressure (DBP) ≥ 85 mmHg or current use of antihypertensive drugs, (3) fasting TG level ≥ 150 mg/dL or current use of any lipid-lowering agents, (4) HDL-C level < 40 mg/dL (men) or 50 mg/dL (women), and (5) fasting serum glucose level ≥ 100 mg/dL or current use of anti-diabetic medication. The metabolically unhealthy group (MUH) included individuals diagnosed with MetS, while the metabolically healthy group (MH) did not.

BMI was categorized into normal-weight, overweight, and obesity^13^: normal-weight (NW), 18.5 kg/m^2^ ≤ BMI < 23 kg/m^2^; overweight (OW), 23 kg/m^2^ ≤ BMI < 25 kg/m^2^; obese (O), BMI ≥ 25 kg/m^2^. By combining MetS and BMI categories, all participants were assigned to one of the following six groups: MHNW, metabolically healthy and normal weight; MHO, metabolically healthy and overweight; MHO, metabolically healthy and obese; MUHNW, metabolically unhealthy and normal weight; MUHOW, metabolically unhealthy and overweight; MUHO, metabolically unhealthy and obese.

The NHSPs collected information regarding hypertension, family history of diabetes, smoking status, alcohol consumption, and physical activity from self-reported questionnaires. Smoking status was categorized as never smoker, former smoker, or current smoker. Never smokers were defined as individuals who answered “No” to “Have you ever smoked five packs (100 cigarettes) or more in your lifetime?” Former smokers were defined as individuals who answered “Yes, but I quit” to this question. Current smokers were defined as individuals who answered "Yes, and I currently smoke cigarettes." Alcohol consumption was classified as rare (less than two drinks per week for men, less than one drink per week for women), moderate (not meeting the definition of the 'rare' nor 'heavy' categories), or heavy (14 or more drinks per week for men, seven or more drinks per week for women)^14^. Physical activity was divided into three categories: low (not meeting the definition of the ‘moderate’ nor ‘high’ categories), moderate (five or more days of moderate-intensity exercises per week; five or more days of combination of moderate-intensity or vigorous-intensity exercises per week), and high (three or more days of vigorous-intensity exercises per week; seven or more days of combination of moderate-intensity or vigorous-intensity exercises per week)^15^. We categorized economic status into three groups by income-based insurance premium: low, 1–3rd deciles; middle, 4th–7th deciles; and high, 8th–10th deciles. Residential areas were categorized using residential area codes for metropolitan areas and other regions. Seven cities were classified as metropolitan areas by adding Seoul, a special city, to the six metropolitan cities of Incheon, Daejeon, Gwangju, Daegu, Ulsan, and Busan. Metropolitan cities have populations of 1 million or more, most of the region is a city, and more than 60% of the population is densely populated within the city.

Hypertension, diabetes mellitus (DM), and dyslipidemia were defined as "yes" or "no" based on (1) a self-reported questionnaire at the baseline NHSPs, or (2) any prescription of antihypertensives (angiotensin-converting enzyme inhibitors, angiotensin receptor blockers, calcium channel blockers, beta-blockers, alpha-blockers, diuretics, other vasodilators, and/or their combinations), glucose-lowering agents (metformin, sulfonylurea, thiazolidinedione, inhibitor of dipeptidyl peptidase 4, sodium-glucose cotransporter 2 inhibitor, insulin, glucagon-like peptide 1 receptor agonist, and/or their combinations), and anti-dyslipidemic drugs (statin, omega-3 fatty acids, niacin, or bile acid sequestrants) for 90 days or longer during 2009 and 2010, respectively.

### Outcomes and study duration

The endpoint of this study was to compare the occurrence rates of CVDs and all-cause mortality in the metabolic healthiness and obesity groups after enrollment (2009–2010). The composite outcome is sum of all-cause mortality and incidence of CVDs. CVDs were defined when the main diagnosis (I20-I25 or I60-I69) was recorded at least twice in outpatients or once in hospitalized patients. CVDs included IHD (I20-I25) and CbVDs (I60-I69) based on ICD-10 codes. CbVDs were further divided into ischemic, hemorrhagic, and other CbVDs according to the diagnosis code as follows: ischemic CbVDs were coded as I63 (cerebral infarction), I65 (occlusion and stenosis of precerebral arteries, not resulting in cerebral infarction), and I66 (occlusion and stenosis of cerebral arteries, not resulting in cerebral infarction); hemorrhagic CbVDs as I60 (subarachnoid hemorrhage), I61 (intracranial hemorrhage), and I62 (other nontraumatic intracranial hemorrhage); and other CbVDs as I64 (stroke, not specified as hemorrhage or infarction), I67 (other cerebrovascular diseases), I68 (cerebrovascular disorders in diseases classified elsewhere), and I69 (sequelae of cerebrovascular disease). We conducted subgroup analyses for each IHDs, CbVDs, and all-cause mortality.

The start date of the research was defined as the day of the first health examination between 2009 and 2010. For participants diagnosed with CVD between 2011 and 2019, the research end date was the date of initial diagnosis of the disease. In cases where the participant died before a diagnosis of diabetes was made, the end date was defined as the date of death. Similarly, in cases where the participants had not died or had not been diagnosed with diabetes during the study period, the end date was the latest date of the last outpatient clinic visit, last health screening, or last when the participants took the prescribed medication.

### Statistical analysis

Continuous variables are presented as mean ± standard deviation (SD). Categorical variables were presented as number and percentage of participants (%). Analysis of variance (ANOVA) tests for continuous variables and chi-squared tests for categorical variables were used to check for group differences. To investigate the association between MetS, obesity, and composite outcomes (all-cause mortality and incidence of CVDs), outcome-free survival rates were estimated and compared using the Kaplan–Meier method and log-rank test. Hazard ratios (HRs) and 95% confidence intervals (CIs) were calculated to examine the association between MetS, obesity, and the composite outcomes based on Cox proportional hazards regression models after adjusting for confounders. We built three Cox proportional hazard regression models after adjusting for age, smoking status, alcohol consumption status, physical activity, economic status, residence area, alanine aminotransferase (ALT), and gamma-glutamyl transferase (GGT). We performed subgroup analysis for each outcome (IHDs, CbVDs including ischemic and hemorrhagic CbVD, and all-cause deaths). The outcome-free survival rates were estimated using the Kaplan–Meier method. The HRs and 95% CIs for each outcome were calculated using the Cox proportional hazards regression model identical to the composite outcome.

All p values were two-sided, and statistical significance was set at *p* < 0.05. Statistical analyses were performed using the statistical package SAS enterprise version 7.1 (SAS Inc., Cary, NC, USA).

### Ethics approval and consent to participate

Not applicable. The ethics committee of National Health Insurance Service (NHIS) waived the need for informed consent because the data from the NHIS-HEALS were anonymized at all stages, including during data cleaning and statistical analysis. The Institutional Review Board of the Chungbuk National University Hospital approved the present study (CBNUH-2022-01-039).

## Results

A total of 151,706 participants (85,249 men and 66,457 women) included in this study, and the median follow-up duration was 9.7 years (minimum 33 days, maximum 4105 days). Table 1 shows the baseline characteristics of the study population according to the combination of MetS and BMI. All covariates were significantly different among the six groups (*p* < 0.001). (Table 1) Within the same BMI category, the MH group was younger than the MUH group was. Waist circumference, SBP, fasting glucose, ALT, and GGT levels were lower in the MH group than in the MUH group. In both sexes, TG levels were higher in the MUH groups than the MH groups, while HDL-C and LDL-C levels tended to be higher in the MH groups than the MUH groups. The proportion of current smokers was lower in the MH group than in the MUH group in both sexes. Among men, the MH group drank less alcohol and engaged in more regular physical activity than the MUH group. Economic status was higher in the male MH group. However, females in the MUH group drank less alcohol and had a higher economic status than those in the MH group.

**Table 1:** Baseline characteristics of study participants according to metabolic healthy and obesity.

	MHNW	MHOW	MHO	MUHNW	MUHOW	MUHO	*p*-value
**Men**							
Number	23,944	18,344	14,498	4046	7424	16,993	
Age, years	57.7 ± 8.9	56.3 ± 7.9	55.5 ± 7.3	59.8 ± 9.2	58.5 ± 8.5	57.2 ± 7.9	< 0.001
Body mass index, kg/m^2^	21.4 ± 1.2	24.0 ± 0.6	26.5 ± 1.4	21.7 ± 1.0	24.1 ± 0.6	27.3 ± 1.9	< 0.001
Waist circumference, cm	78.4 ± 5	83.6 ± 4.1	87.9 ± 5.0	81.6 ± 5.3	86.3 ± 4.9	92.6 ± 5.6	< 0.001
Systolic blood pressure, mmHg	122.6 ± 14.7	124.3 ± 14.1	125.6 ± 13.7	132.3 ± 14.4	132.0 ± 13.8	132.4 ± 14.1	< 0.001
Fasting glucose, mg/Dl	98.2 ± 23.4	98.6 ± 21.2	97.6 ± 19.9	123.4 ± 40.4	118.9 ± 36.2	115.6 ± 32.6	< 0.001
Total cholesterol, mg/dL	192.3 ± 33.5	198.4 ± 34.2	200.1 ± 34.1	194.7 ± 42.2	198.0 ± 39.0	200.0 ± 38.2	< 0.001
Triglyceride, mg/Dl	113.5 ± 68.3	131.3 ± 76.1	135.9 ± 76.4	205.4 ± 118.0	213.1 ± 117.4	209.2 ± 119.4	< 0.001
HDL cholesterol, mg/dL	57.0 ± 29.1	53.6 ± 25.1	52.9 ± 26.3	48.3 ± 24.6	47.1 ± 23.9	47.5 ± 22.2	< 0.001
LDL cholesterol, mg/dL	114.2 ± 35.9	119.7 ± 34.7	121.6 ± 36.9	106.4 ± 42.3	109.7 ± 39.6	111.9 ± 40.5	< 0.001
ALT, mg/dL	22.9 ± 15.9	25.8 ± 17.1	29.7 ± 19.7	28.1 ± 20.0	30.2 ± 21.7	35.2 ± 22.2	< 0.001
GGT, mg/dL	40.6 ± 57.4	43.1 ± 47.6	47.5 ± 45.0	70.1 ± 108.8	61.1 ± 75.5	64.9 ± 66.3	< 0.001
**Smoking, n (%)**							< 0.001
Never	8480 (35.4)	6731 (36.7)	5446 (37.6)	1245 (30.8)	2469 (33.3)	5819 (34.2)	
Ex-smoker	6715 (28.0)	6314 (34.4)	5107 (35.2)	1173 (29.0)	2529 (34.1)	6104 (35.9)	
Current smoker	8749 (36.5)	5299 (28.9)	3945 (27.2)	1628 (40.2)	2426 (32.7)	5070 (29.8)	
**Alcohol consumption, n (%)**							< 0.001
Rare	11,218 (46.9)	7855 (42.8)	6056 (41.8)	1680 (41.5)	3023 (40.7)	6521 (38.4)	
Moderate	11,968 (50.0)	9726 (53.0)	7673 (52.9)	2179 (53.9)	4014 (54.1)	9299 (54.7)	
Heavy	758 (3.2)	763 (4.2)	769 (5.3)	187 (4.6)	387 (5.2)	1173 (6.9)	
**Physical activity, n (%)**							< 0.001
Low	18,070 (75.5)	13,115 (71.5)	10,246 (70.7)	3041 (75.2)	5430 (73.1)	12,361 (72.7)	
Moderate	824 (3.4)	701 (3.8)	537 (3.7)	156 (3.9)	260 (3.5)	617 (3.6)	
High	5050 (21.1)	4528 (24.7)	3715 (25.6)	849 (21.0)	1734 (23.4)	4015 (23.6)	
**Economic status, n (%)**							< 0.001
Low	3972 (16.6)	2607 (14.2)	2042 (14.1)	735 (18.2)	1286 (17.3)	2831 (16.7)	
Middle	7762 (32.4)	5190 (28.3)	4076 (28.1)	1349 (33.4)	2284 (30.8)	5187 (30.5)	
High	12,210 (51.0)	10,547 (57.5)	8380 (57.8)	1962 (48.5)	3854 (51.9)	8975 (52.8)	
**Area of residence, n (%)**							< 0.001
Metropolitan city	10,951 (45.7)	8453 (46.1)	6400 (44.1)	1826 (45.1)	3461 (46.6)	7364 (43.3)	
Other area	12,993 (54.3)	9891 (53.9)	8098 (55.9)	2220 (54.9)	3963 (53.4)	9629 (56.7)	
Diabetes, n (%)	1666 (7.0)	1249 (6.8)	777 (5.4)	1297 (32.1)	2094 (28.2)	4213 (24.8)	< 0.001
Hypertension, n (%)	5045 (21.1)	4771 (26.0)	4115 (28.4)	2272 (56.2)	4245 (57.2)	10,213 (60.1)	< 0.001
Dyslipidemia, n (%)	1373 (5.7)	1338 (7.3)	1102 (7.6)	1183 (29.2)	2177 (29.3)	4890 (28.8)	< 0.001
**Women**							
Number	20,878	12,850	10,784	4244	5313	12,388	
Age, years	57.3 ± 8.6	57.7 ± 8.0	58.2 ± 8.0	63.9 ± 9.5	63.0 ± 8.8	62.4 ± 8.6	< 0.001
Body mass index, kg/m^2^	21.3 ± 1.1	23.9 ± 0.6	26.8 ± 1.7	21.6 ± 1.1	24.0 ± 0.6	27.8 ± 2.4	< 0.001
Waist circumference, cm	72.8 ± 5.3	78.0 ± 4.7	83.1 ± 5.9	76.6 ± 6.0	81.8 ± 5.4	88.5 ± 6.4	< 0.001
Systolic blood pressure, mmHg	119.0 ± 15.0	121.5 ± 14.9	124.0 ± 14.9	130.5 ± 15.1	131.0 ± 14.9	132.2 ± 15.1	< 0.001
Fasting glucose, mg/dL	93.3 ± 17.3	93.7 ± 16.4	93.9 ± 16.0	112.3 ± 32.6	111.4 ± 32.0	110.6 ± 29.9	< 0.001
Total cholesterol, mg/dL	202.9 ± 35.2	207.1 ± 36.5	209.3 ± 36.3	204.5 ± 41.8	206.6 ± 40.9	209.3 ± 41.1	< 0.001
Triglyceride, mg/dL	101.6 ± 56.6	110.5 ± 59.7	112.0 ± 57.1	175.3 ± 93.1	175.9 ± 90.7	172.8 ± 92.4	< 0.001
HDL cholesterol, mg/dL	62.1 ± 36.3	60.1 ± 32.2	60.3 ± 34.3	48.8 ± 20.2	49.9 ± 25.9	51.0 ± 22.0	< 0.001
LDL cholesterol, mg/dL	122.9 ± 35.3	126.9 ± 36.6	129.1 ± 38.3	120.8 ± 39.0	122.7 ± 39.4	124.6 ± 40.4	< 0.001
ALT, mg/dL	19.2 ± 14.3	21.2 ± 15.9	23.2 ± 17.6	21.9 ± 17.9	23.9 ± 14.4	27.7 ± 21.0	< 0.001
GGT, mg/dL	20.2 ± 21.4	22.1 ± 21.0	24.0 ± 24.1	24.7 ± 30.9	26.6 ± 23.8	31.1 ± 34.0	< 0.001
**Smoking, n (%)**							< 0.001
Never	20,385 (97.6)	12,609 (98.1)	10,596 (98.3)	4109 (96.8)	5181 (97.5)	12,123 (97.9)	
Ex-smoker	146 (0.7)	83 (0.6)	54 (0.5)	34 (0.8)	43 (0.8)	75 (0.6)	
Current smoker	347 (1.7)	158 (1.2)	134 (1.2)	101 (2.4)	89 (1.7)	190 (1.5)	
**Alcohol consumption, n (%) **							< 0.001
Rare	18,413 (88.2)	11,240 (87.5)	9506 (88.1)	3944 (92.9)	4957 (93.3)	11,254 (90.8)	
Moderate	2181 (10.4)	1413 (11.0)	1071 (9.9)	251 (5.9)	293 (5.5)	926 (7.5)	
Heavy	284 (1.4)	197 (1.5)	207 (1.9)	49 (1.2)	63 (1.2)	208 (1.7)	
**Physical activity, n (%) **							< 0.001
Low	16,664 (79.8)	10,082 (78.5)	8604 (79.8)	3495 (82.4)	4231 (79.6)	10,235 (82.6)	
Moderate	739 (3.5)	459 (3.6)	380 (3.5)	118 (2.8)	196 (3.7)	365 (2.9)	
High	3475 (16.6)	2309 (18.0)	1800 (16.7)	631 (14.9)	886 (16.7)	1788 (14.4)	
**Economic status, n (%) **							< 0.001
Low	5401 (25.9)	3227 (25.1)	2787 (25.8)	1043 (24.6)	1310 (24.7)	3145 (25.4)	
Middle	7225 (34.6)	4589 (35.7)	3946 (36.6)	1435 (33.8)	1797 (33.8)	4515 (36.4)	
High	8252 (39.5)	5034 (39.2)	4051 (37.6)	1766 (41.6)	2206 (41.5)	4728 (38.2)	
**Area of residence, n (%)**							< 0.001
Metropolitan city	9524 (45.6)	5756 (44.8)	4624 (42.9)	1766 (41.6)	2208 (41.6)	4858 (39.2)	
Other area	11,354 (54.4)	7094 (55.2)	6160 (57.1)	2478 (58.4)	3105 (58.4)	7530 (60.8)	
Diabetes, n (%)	830 (4.0)	470 (3.7)	368 (3.4)	1291 (30.4)	1463 (27.5)	3232 (26.1)	< 0.001
Hypertension, n (%)	4160 (19.9)	3196 (24.9)	3288 (30.5)	2678 (63.1)	3474 (65.4)	8540 (68.9)	< 0.001
Dyslipidemia, n (%)	2102 (10.1)	1645 (12.8)	1195 (11.1)	1698 (40.0)	2160 (40.7)	4792 (38.7)	< 0.001

Values are presented as n (%) or mean ± standard deviation.

*MHNW* metabolically healthy normal weight, *MHOW* metabolically healthy overweight, *MHO* metabolically healthy obese, *MUHNW* metabolically unhealthy normal weight, *MUHOW* metabolically unhealthy overweight, *MUHO* metabolically unhealthy obese, *HDL* high-density lipoprotein, *LDL* low-density lipoprotein, *ALT* alanine aminotransferase, *GGT* gamma-glutamyl transferase.

The incidence of DM, hypertension, and dyslipidemia was higher in the MUH group than in the MH group. Within each MH and MUH group, the more obese groups had higher SBP, total cholesterol, LDL-cholesterol, and ALT levels.

**Figure 2:**
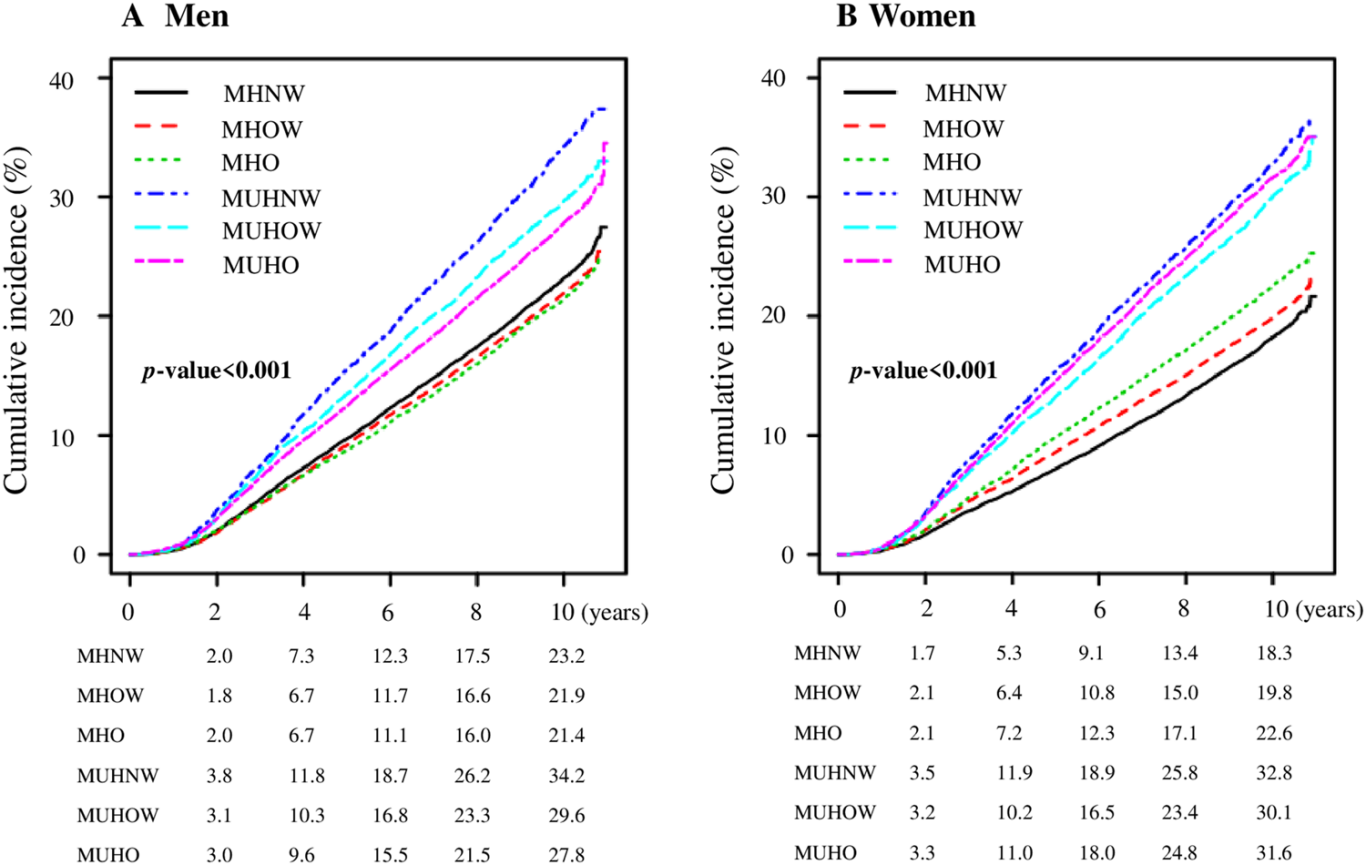
Cumulative incidents of composite outcome (all-cause mortality and incidence of cardiovascular diseases) according to metabolic healthy and obesity.

Figure 2 shows the estimated cumulative incidence of the composite outcomes based on the Kaplan–Meier survival curve. The cumulative incidence of composite outcomes was highest in MUHNW in both sexes, lowest in MHO in men, and lowest in MHNW in women (log-rank test *p* < 0.001). A total of 36,465 composite outcomes were observed, accounting for 24.0% of the total participants (24.4% of men and 23.5% of women) (Supplementary Table 1). At the end of the follow-up period, the estimated cumulative incidences of composite outcomes were as follows: MHNW 23.0%, MHOW 21.7%, MHO 21.3%, MUHNW 34.0%, MUHOW 29.5%, and MUHO 27.6%, respectively, in men, and MHNW 18.0%, MHOW 19.8%, MHO 22.5%, MUHNW 32.9%, MUHOW 30.0%, and MUHO 31.7%, respectively, in women.

**Figure 3:**
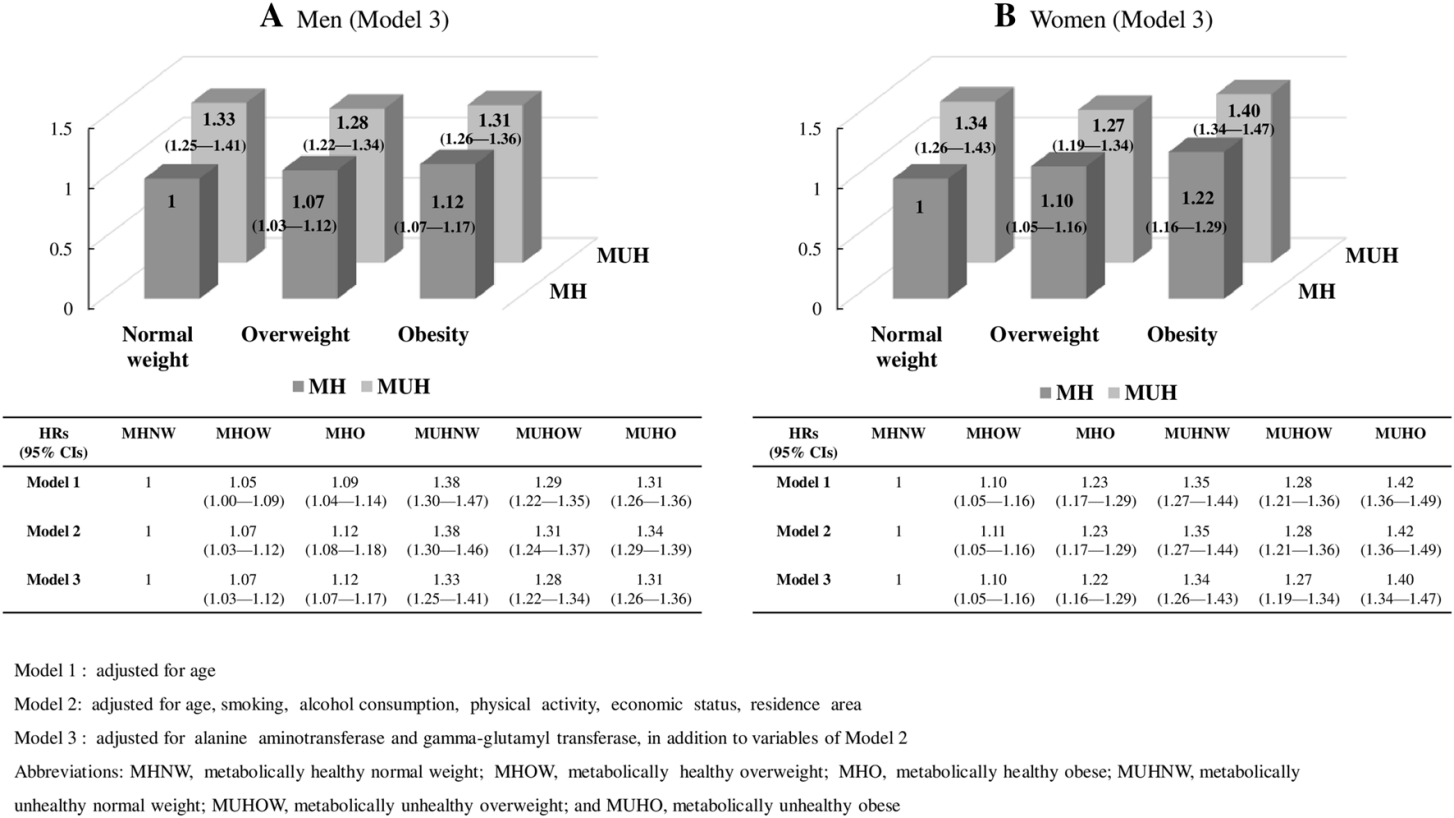
Cox proportional hazards regression models for composite outcome (all-cause mortality and incidence of cardiovascular diseases).

Figure 3 presents the results of the Cox proportional hazard regression models to examine the association between MetS, BMI category, and the incidence of composite outcomes. Compared to MHNW, HRs (95% CIs) for the composite outcome of MHOW, MHO, MUHNW, MUHOW, and MUHO were 1.07 (1.03–1.12), 1.12 (1.07–1.17), 1.33 (1.25–1.41), 1.28 (1.22–1.34), and 1.31 (1.26–1.36), respectively, in men, and 1.10 (1.05–1.16), 1.22 (1.16–1.29), 1.34 (1.26–1.43), 1.27 (1.19–1.34), and 1.40 (1.34–1.47), respectively, in women after adjusting for age, smoking status, alcohol consumption, physical activity, economic status, residence area, ALT, and GGT levels (Model 3). In the metabolically healthy group, the higher BMI group had the higher risk of composite outcomes. The metabolically unhealthy group had a higher risk in any given BMI group.

**Figure 4:**
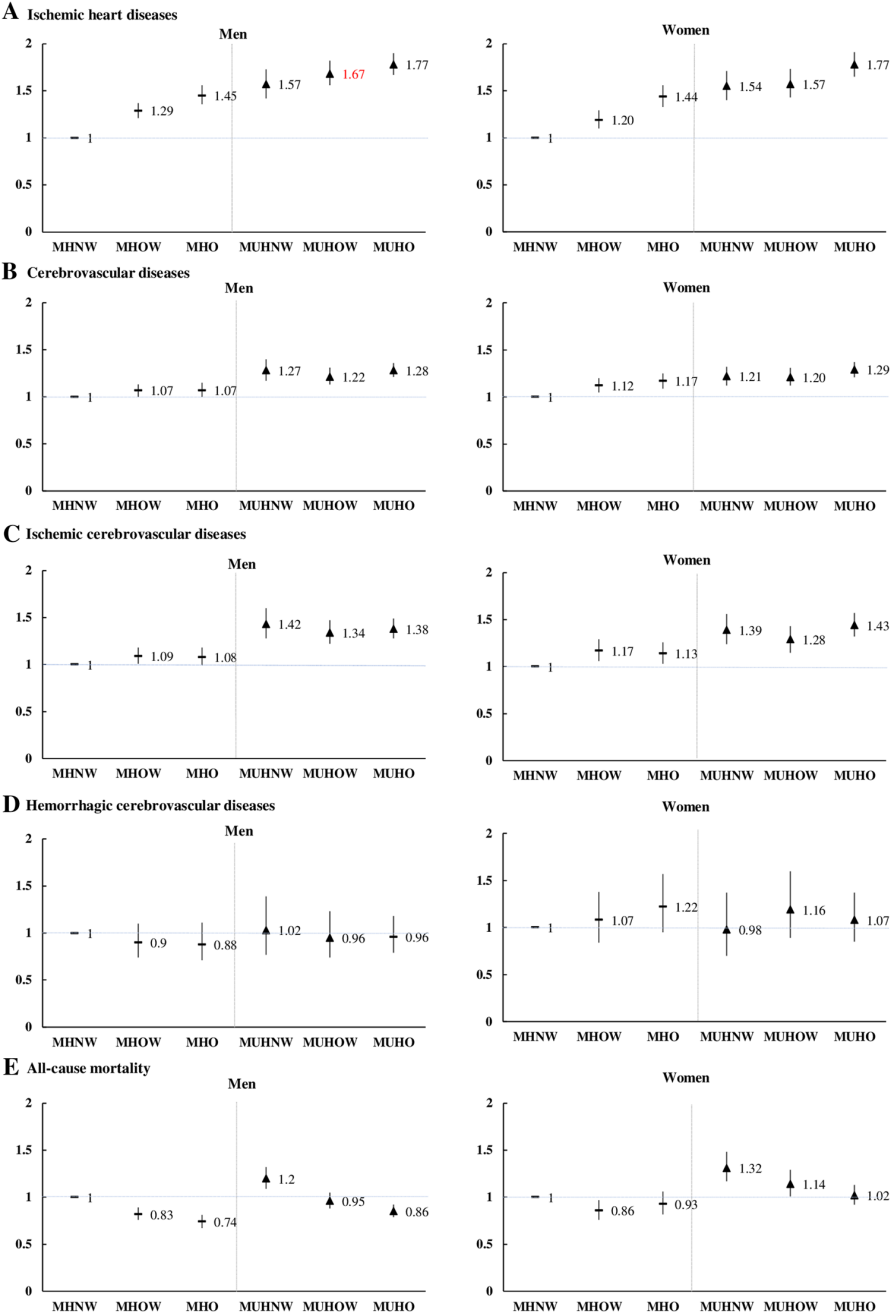
Full-adjusted Cox proportional hazards regression models for incidence of ischemic heart diseases, cerebrovascular diseases, and all-cause mortality.

Subgroup analysis was conducted to investigate the association between MetS, BMI category, and each outcome (IHDs, CbVDs, ischemic CbVD, hemorrhagic CbVD, and all-cause mortality) (Fig. 4). Compared to MHNW, fully adjusted HRs (95% CIs) for IHDs of MHOW, MHO, MUHNW, MUHOW, and MUHO were 1.29 (1.21–1.37), 1.45 (1.36–1.56), 1.57 (1.42–1.73), 1.67 (1.54–1.80), and 1.77 (1.66–1.88), respectively, in men, and 1.20 (1.10–1.30), 1.44 (1.33–1.56), 1.54 (1.39–1.71), 1.57 (1.43–1.72), and 1.77 (1.64–1.90), respectively, in women. Fully adjusted HRs (95% CIs) for CbVDs of MHOW, MHO, MUHNW, MUHOW, and MUHO, compared to MHNW, were 1.06 (1.00–1.13), 1.07 (1.01–1.15), 1.27 (1.16–1.39), 1.22 (1.13–1.31), and 1.28 (1.21–1.36), respectively, in men, and 1.12 (1.05–1.20), 1.17 (1.09–1.25), 1.21 (1.11–1.32), 1.20 (1.11–1.30), and 1.29 (1.21–1.37), respectively, in women. Fully adjusted HRs (95% CIs) for ischemic CbVDs of MHOW, MHO, MUHNW, MUHOW, and MUHO were 1.09 (1.01–1.18), 1.08 (0.99–1.18), 1.42 (1.28–1.59), 1.34 (1.22–1.47), and 1.38 (1.28–1.49), respectively, in men, and 1.17 (1.06–1.29), 1.13 (1.03–1.25), 1.39 (1.24–1.56), 1.28 (1.15–1.43), and 1.43 (1.32–1.56), respectively, in women. The risk of hemorrhagic CbVDs was not significantly associated with the six MetS and BMI categories combined. Compared to MHNW, HRs (95% CIs) for all-cause mortality of MHOW, MHO, MUHNW, MUHOW, and MUHO were 0.83 (0.77–0.89), 0.74 (0.67–0.81), 1.20 (1.09–1.32), 0.95 (0.87–1.04), and 0.86 (0.79–0.92), respectively, in men, and 0.86 (0.76–0.98), 0.93 (0.82–1.06), 1.32 (1.17–1.49), 1.14 (1.00–1.29), and 1.02 (0.92–1.13), respectively, in women after full adjustment.

Apart from to Model 3, additional analysis was performed to check the marginal effect, which is how the composite outcome and each outcome change when the degree of obesity changes in the MH group and the MUH group (Model 4, Supplementary Table 2). Compared to the MH, the HRs (95% Cis) of the MUH for the composite outcomes were 1.33 (1.25–1.41) and 1.34 (1.26–1.43) in men and women, respectively. In the MH group, the HRs increased according to the increase in BMI (overweight in MH, 1.07 [1.03–1.12] in men and 1.10 [1.05–1.16] in women; obese in MH, 1.12 [1.07–1.17] in men and 1.22 [1.16–1.29] in women), but in the MUH group, the HRs in the overweight and obese groups decreased significantly compared to the MUHNW group (MUH*Overweight, 0.90 [0.83–0.97] in men and 0.86 [0.79–0.94] in women; MUH*Obese, 0.88 [0.81–0.94] in men and 0.86 [0.79–0.93] in women).

## Discussion

Based on the Korean NHIS-HEALS data, this retrospective study demonstrated that the risk of composite outcome increased in the MHOW, MHO, and MUH groups compared to the MHNW group. In particular, after stratifying the composite outcome into IHD, CbVD, and all-cause death, all MUH groups showed an increased risk of IHD, CbVD, and ischemic CbVD incidence in both sexes compared to the MHNW group.

Recently, a few studies have shown that MHO did not increase the risk of CVD incidence more than MHNW^16^; however, in our research, the risk of composite outcome, IHDs, CbVDs, and ischemic CbVDs in MHO was higher in MHNW. All-cause mortality is lower in MHO than in MHNW in men but not significantly different between the two groups in women. The risk of all-cause mortality increased even in MUHNW in both sexes.

In many cases, MetS and obesity coexist, and both can contribute to CVD^17^. Accordingly, through the results from analyses of the marginal effect conducted to confirm the interaction between MetS and obesity, the HRs for the composite outcome of the MUH group was found to be approximately 1.3-fold higher than those of the MH group (*p* < 0.001) (Supplementary Table 2). Interestingly, the effect of BMI on the composite outcome was different in direction between the MH and MUH groups (*p* < 0.01). In the MH group, the overweight and obese groups had a higher risk in all outcomes, except hemorrhagic CbVDs and all-cause death, than the normal-weight MH group (*p* < 0.01). These results are not significantly different from those of previous studies^18–20^; the CVD risk in the MUH group increased by 1.5 to 2.0 times compared to the MH group, and the CVD risk in the overweight or obesity group rose by 1.2 to 1.6 times compared to the normal-weight group.

In the MH group, the risk of composite outcome increased with the BMI increased, whereas in the MUH group, the risk of the overweight and obesity group showed a decreased risk of the composite outcome and IHD (interaction *p* < 0.05). Given these results, although additional research is needed, the effects of obesity on CVD outcomes may vary depending on metabolic health.

Previous studies have shown that various factors influence the occurrence of the MUH phenotype, and age, alcohol consumption status, low level of physical activity, low education level, and smoking are thought to be factors^21^. In addition, compared with MUHO, the MHO group had a better quality diet with a high intake of fruits, whole grains, meat, and beans^21^.

In our study, men showed similar results. In contrast, among women, the MH group showed a higher percentage of moderate alcohol consumption and lower economic status, and the MUH group had a relatively healthier lifestyle. These differences might be due to the MUH group's chance for early detection and treatment of the disease through regular check-ups or hospital visits according to the higher economic status and the possibility of healthy lifestyle changes to manage chronic conditions, such as refraining from alcohol consumption.

However, the mechanism by which metabolic unhealthiness or obesity influences CVD and death has not been elucidated. In a study by Kassi et al., the group with metabolic syndrome showed high proinflammatory or inflammatory marker levels, and inflammation caused ApoA-I and HDL dysfunction, suggesting that it could be related to the development of CVD or type 2 DM, in addition to insulin resistance^22^. Differences in fat distribution can also be explained as the cause of worse CVD outcomes in an MUH population. In the case of the MUHNW population, there is little adipose tissue in the gluteo-femoral region that can store excess fat. Instead, as trunk fat mass increases, previous studies have asserted that CVD risk increases independently^23^. In addition, recently, Single nucleotide polymorphisms (SNPs) related to lipid metabolism or insulin or glucose metabolism were observed in the MUHO or MUHNW groups, suggesting that they may be associated with CVD outcome at the gene level and the phenotype of obesity or MUH^23,24^.

In our study, the risk of all-cause mortality tended to decrease with increasing BMI in both sexes in MH groups (Overweight in MH, 0.83 [0.77–0.89] in men and 0.86 [0.76–0.98] in women; Obese in MH, 0.74 [0.67–0.81] in men and 0.93 [0.82–1.06] in women) (Supplementary Table 2). Although not statistically significant, this trend is also seen in the MUH groups. Similar to other previous studies^25,26^, this is thought to be a result in support of the “obesity paradox,” in which all-cause mortality decreases with increasing BMI.

Although unclear, this “obesity paradox” is sometimes explained due to earlier access to medical treatment in obese populations and the benefits of higher metabolic reserves^26^. Recently, particularly in MHO, a mechanism has been suggested that intrinsic healthy adipose tissue allows excess adiposity without adipocyte dysfunction. Intrinsic healthy adipose tissue molecular action is linked with efficient fat storage and lipid droplet formation, high adipogenesis capacity, low extracellular matrix fibrosis, angiogenesis potential, adipocyte browning, and low macrophage infiltration/activation^27^.

However, there is a limitation in that the cohort data used in this study did not include data on inflammatory markers such as interleukin-6 and high-sensitivity C-reactive protein, muscle mass, body fat distribution, diet, and SNP. Therefore, it was impossible to confirm a direct relationship between the following mechanisms and the results.

There was an age difference between each group of at least 1.7 to 5.7 years. Even though age was adjusted in all models, the residual effect of age-related hormonal change, metabolic derangement, and other risk factors should be considered as potential limitations when interpreting this study. In addition, since we did not consider the contribution of each factor involved in metabolic unhealthiness to the CVD outcome, the actual risk may differ from these results. Furthermore, both MetS and obesity are likely to be transient at one point in time. This study estimated the risk of disease incidence and death according to MetS and obesity only at baseline. The possibility of risk changes according to status changes was not reflected due to cohort data limitations. Some previous studies^28,29^ showed that elevated fasting glucose and low HDL-C were associated with increased mortality, but the results did not include changes in the overall observation period.

Since the definition of MetS or metabolic unhealthiness is not yet clear, even in previous studies, each researcher conducted the analysis using different criteria. The results also differed depending on the criteria used. However, in our study, metabolic unhealthiness was defined by applying criteria for MetS that are familiar to the clinical field. It has the strength of analyzing a long-term follow-up period of approximately 10 years for a group that can relatively represent Koreans.

In addition, this study has the strength of subdividing and comparing groups according to the metabolic health of each obesity degree and additionally analyzing the interaction between obesity and metabolic health.

This study confirmed that metabolically unhealthy and increased BMI at a single time point could also affect the risk of CVD and death. Significantly, the risk of CVD and all-cause mortality was higher in metabolically unhealthy individuals with BMI within the normal range than in other groups. Efforts in the clinical field are necessary for disease prevention and management.

## Conclusion

For composite outcome, high BMI and metabolic unhealthiness were associated with increased risk. However, in a specific risk of all-cause death result showed an inconsistency direction with the risk of CVD incidence. In the same BMI group, the metabolically unhealthy group had a higher risk of all outcomes than the metabolically healthy group. Even in the metabolically healthy group, the overweight or obese group had a higher risk of the composite outcome and CVD incidence than the normal weight group. Interestingly, patients with normal weight, if the metabolism is unhealthy, have an even higher risk of CVD and all-cause death than metabolically healthy obese patients, so attention should be paid to prevention.

## Supplementary Information

Supplementary Table 1.

Supplementary Table 2.

## Data Availability

The data that support the findings of this study are available from National Health Insurance Sharing Service but restrictions apply to the availability of these data, which were used under license for the current study, and so are not publicly available. Data are however available from the authors upon reasonable request and with permission of National Health Insurance Sharing Service.

## Abbreviations

CVD: Cardiovascular disease
MetS: Metabolic syndrome
BMI: Body mass index
MHNW: Metabolically healthy normal weight
MHOW: Metabolically healthy overweight
MHO: Metabolically healthy obese
MUHNW: Metabolically unhealthy normal weight
MUHOW: Metabolically unhealthy overweight
MUHO: Metabolically unhealthy obese
HR: Hazard ratios
CI: Confidence interval
IHD: Ischemic heart disease
NCDs: Non-communicable diseases
HDL-C: High-density lipoprotein cholesterol
LDL-C: Low-density lipoprotein cholesterol
NHSPs: National Health Screening Program
NHIS: National Health Insurance Service
HEALS: Health Screening
CbVDs: Cerebrovascular diseases
SBP: Systolic blood pressure
DBP: Diastolic blood pressure
DM: Diabetes mellitus
SD: Standard deviation
ANOVA: Analysis of variance
ALT: Alanine aminotransferase
GGT: Gamma-glutamyl transferase
SNP: Single nucleotide polymorphism
