# Author Correction: Effect of metabolic health and obesity on all-cause death and CVD incidence in Korean adults: a retrospective cohort study

**DOI:** 10.1038/s41598-023-29688-5

**Published:** 2023-02-15

**Authors:** Ye-Seul Kim, Sang-Jun Shin, Yonghwan Kim, Joungyoun Kim, Hee-Taik Kang

**Affiliations:** 1 Department of Family Medicine, Chungbuk National University Hospital, 776 1-Soonwhan-ro, Seowon-gu, Cheongju, 28644 Republic of Korea; 2 Biostatistics Collaboration Unit, Department of Biomedical Systems Informatics, Yonsei University College of Medicine, 50-1 Yonsei-ro, Seodaemun-gu, Seoul, 03722 Republic of Korea; 3 College of Nursing, Mo-Im Kim Nursing Research Institute, Yonsei University, 50-1, Yonsei-ro, Seodaemun-gu, Seoul, 03722 Republic of Korea; 4 Department of Artificial Intelligence, University of Seoul, 163 Seoulsiripdae-ro, Dongdaemun-gu, Seoul, 02504 Republic of Korea; 5 Department of Family Medicine, Chungbuk National University College of Medicine, 1 Chungdae-ro, Seowon-gu, Cheongju, 28644 Chungbuk Republic of Korea

Correction to: *Scientific Reports* 10.1038/s41598-022-27097-8, published online 12 January 2023

The original version of the Article contained an error in the Author Information section.

“These authors contributed equally: Ye-Seul Kim, Sang-Jun Shin, Joungyoun Kim and Hee-Taik Kang.”

now reads:

“These authors contributed equally: Ye-Seul Kim and Sang-Jun Shin.”

The original Article has been corrected.

