# Supplementary material for: Effect of metabolic health and obesity on all-cause death and CVD incidence in Korean adults: a retrospective cohort study
Source: Sci Rep. 2023 Jan 12;13:639. doi: 10.1038/s41598-022-27097-8 (PMC9837041; doi:10.1038/s41598-022-27097-8)
Supplement: Supplementary file 1 — Supplementary Table 1. [file 41598_2022_27097_MOESM1_ESM.docx]

| **Men** | Sample size | **Composite Outcome** | | | **All-cause death** | | |
| --- | --- | --- | --- | --- | --- | --- | --- |
|  |  | Number of  events (%) | Person-years | Incidence rate per 1,000 person-year | Number of  events (%) | Person-years | Incidence rate per 1,000 person-year |
| **MHNW** | 23,944 | 5,501 (23.0) | 213,186.6 | 25.8 | 2,053 (8.6) | 229,257.2 | 9.0 |
| **MHOW** | 18,344 | 3,982 (21.7) | 164,061.3 | 24.3 | 996 (5.4) | 177,776.8 | 5.6 |
| **MHO** | 14,498 | 3,087 (21.3) | 130,051.6 | 23.7 | 605 (4.2) | 141,060.0 | 4.3 |
| **MUHNW** | 4,046 | 1,375 (34.0) | 34,031.6 | 40.4 | 551 (13.6) | 38,098.8 | 14.5 |
| **MUHOW** | 7,424 | 2,191 (29.5) | 63,753.6 | 34.4 | 640 (8.6) | 71,428.8 | 9.0 |
| **MUHO** | 16,993 | 4,690 (27.6) | 147,429.3 | 31.8 | 1,073 (6.3) | 164,588.8 | 6.5 |

**Supplementary Table 1.** Cumulative number and incidence rate of composite outcome and all-cause death according to BMI and MetS

| **Women** | Sample size | **Composite Outcome** | | | **All-cause death** | | |
| --- | --- | --- | --- | --- | --- | --- | --- |
|  |  | Number of  events (%) | Person-years | Incidence rate per 1,000 person-year | Number of  events (%) | Person-years | Incidence rate per 1,000 person-year |
| **MHNW** | 20,878 | 3,760 (18.0) | 190,754.1 | 19.7 | 737 (3.5) | 204,182.0 | 3.6 |
| **MHOW** | 12,850 | 2,546 (19.8) | 116,345.1 | 21.9 | 359 (2.8) | 126,375.9 | 2.8 |
| **MHO** | 10,784 | 2,422 (22.5) | 96,734.6 | 25.0 | 346 (3.2) | 106,248.6 | 3.3 |
| **MUHNW** | 4,244 | 1,396 (32.9) | 35,913.4 | 38.9 | 421 (9.9) | 40,848.4 | 10.3 |
| **MUHOW** | 5,313 | 1,594 (30.0) | 45,899.2 | 34.7 | 387 (7.3) | 51,776.8 | 7.5 |
| **MUHO** | 12,388 | 3,921 (31.7) | 105,824.0 | 37.1 | 747 (6.0) | 121,288.4 | 6.2 |

**Abbreviations:** MHNW, metabolically healthy normal weight; MHOW, metabolically healthy overweight; MHO, metabolically healthy obese; MUHNW, metabolically unhealthy normal weight; MUHOW, metabolically unhealthy overweight; and MUHO, metabolically unhealthy obese.
