# Supplementary material for: Effect of metabolic health and obesity on all-cause death and CVD incidence in Korean adults: a retrospective cohort study
Source: Sci Rep. 2023 Jan 12;13:639. doi: 10.1038/s41598-022-27097-8 (PMC9837041; doi:10.1038/s41598-022-27097-8)
Supplement: Supplementary file 2 — Supplementary Table 2. [file 41598_2022_27097_MOESM2_ESM.docx]

**Supplementary Table 2.** Cox proportional hazard regression according to independent variable (Model 4)

| **HRs (95% CIs)** | **Composite outcome** | **CVDs** | | | | **All-cause death** |
| --- | --- | --- | --- | --- | --- | --- |
|  |  | **IHDs** | **CbVDs** | **Ischemic CbVDs** | **Hemorrhagic CbVDs** |  |
| **Men** |  |  |  |  |  |  |
| MHNW | 1 | 1 | 1 | 1 | 1 | 1 |
| MUH | 1.33 (1.25—1.41) | 1.57 (1.42—1.73) | 1.27 (1.16—1.39) | 1.42 (1.28—1.59) | 1.02 (0.76—1.38) | 1.20 (1.09—1.32) |
| Overweight in MH | 1.07 (1.03—1.12) | 1.29 (1.21—1.37) | 1.06 (1.00—1.13) | 1.09 (1.01—1.18) | 0.90 (0.74—1.10) | 0.83 (0.77—0.89) |
| Obese in MH | 1.12 (1.07—1.17) | 1.45 (1.36—1.56) | 1.07 (1.01—1.15) | 1.08 (0.99—1.18) | 0.88 (0.70—1.11) | 0.74 (0.67—0.81) |
| MUH*Overweight | 0.90 (0.83—0.97) | 0.83 (0.73—0.94) | 0.90 (0.80—1.01) | 0.86 (0.75—1.00) | 1.04 (0.70—1.56) | 0.97 (0.84—1.11) |
| MUH*Obese | 0.88 (0.81—0.94) | 0.78 (0.70—0.87) | 0.94 (0.84—1.05) | 0.90 (0.78—1.03) | 1.07 (0.73—1.56) | 0.97 (0.84—1.11) |
| **Women** |  |  |  |  |  |  |
| MHNW | 1 | 1 | 1 | 1 | 1 | 1 |
| MUH | 1.34 (1.26—1.43) | 1.54 (1.39—1.71) | 1.21 (1.11—1.32) | 1.39 (1.24—1.56) | 0.98 (0.70—1.38) | 1.32 (1.17—1.49) |
| Overweight in MH | 1.10 (1.05—1.16) | 1.20 (1.10—1.30) | 1.12 (1.05—1.20) | 1.17 (1.06—1.29) | 1.07 (0.83—1.37) | 0.86 (0.76—0.98) |
| Obese in MH | 1.22 (1.16—1.29) | 1.44 (1.33—1.56) | 1.17 (1.09—1.25) | 1.13 (1.03—1.25) | 1.22 (0.95—1.57) | 0.93 (0.82—1.06) |
| MUH*Overweight | 0.86 (0.79—0.94) | 0.85 (0.74—0.98) | 0.88 (0.79—1.00) | 0.79 (0.67—0.93) | 1.11 (0.70—1.75) | 1.00 (0.83—1.21) |
| MUH*Obese | 0.86 (0.79—0.93) | 0.79 (0.70—0.90) | 0.91 (0.81—1.01) | 0.91 (0.79—1.06) | 0.89 (0.59—1.36) | 0.83 (0.70—0.99) |

**Abbreviations:** CVD, cardiovascular disease; IHD, ischemic heart disease; CbVD, cerebrovascular disease; MHNW, metabolically healthy and normal weight; MUH, metabolically unhealthy; MH, metabolically healthy
